# Ultrasensitive (Co)polymers Based on Poly(methacrylamide) Structure with Fining-Tunable pH Responsive Value

**DOI:** 10.3390/molecules23081870

**Published:** 2018-07-27

**Authors:** Haiming Fan, Po Li, Wei Li, Hui Li, Xiaonan Huang

**Affiliations:** 1 Shandong Provincial Key Laboratory of Oilfield Chemistry, School of Petroleum Engineering, China University of Petroleum (East China), Qingdao 266580, China; Haimingfan@126.com (H.F.); lihui0714_upc@163.com (H.L.); 2 Department of Chemistry, Capital Normal University, 105 West 3rd Ring North Rd, Beijing 100048, China; 2150702055@cnu.edu.cn (P.L.); 2120702055@cnu.edu.cn (W.L.)

**Keywords:** fine-tuning, pH responsive, poly(methacrylamide)s, phase transition

## Abstract

Novel pH responsive copolymers with tertiary amine groups were prepared by free radical polymerization with 2-(dialkylamino)ethyl methacrylate monomers. These polymers were pH sensitive with the ability to be responsively fine-tuned in aqueous solution, which was proven through titration, transmittance measurements, and proton nuclear magnetic resonance spectroscopy. The polymers were soluble in water at low pH values, induced by electrostatic repulsion between amine groups, and aggregated above their p*K*_a_ value due to the hydrophobic effect of the alkyls. The pH responsive values were precisely tuned from 7.4 to 4.8 by increasing the hydrophobic monomer ratio. Our work provides a novel approach for the development of ultrasensitive pH-responsive polymers for application in biomedical materials.

## 1. Introduction

The development of tumor-targeted drug delivery systems have attracted increasing interest given their potential to address therapeutic issues in clinical practice [1,2,3,4,5,6,7]. Many studies have focused on designing and improving specific transport and activation of intelligent materials that have stimuli responsive properties [8,9,10,11,12,13]. An interesting aspect of the physiology of tumors is their lower extra-cellular pH (6.5–7.2) compared to the surrounding normal tissues and blood, thus pH can be considered as an ideal trigger for tumor tissues and tumor cells [14]. To target the acidic bio-compartments, several smart materials with pH-responsive properties were designed to increase target efficacy. For instance, the Kobayashi group developed a small molecule system to target the tissue of the mice. This pH of this system can transition across two values of pH value, which means a 200-fold proton concentration change [15,16,17]. Despite these remarkable advances, selective targeting by pH-sensitive materials of different endocytic components is challenging owing to the small pH differences in these compartments, such as early endosomes (5.9–6.2) versus lysosomes (5.0–5.5) [18,19,20].

Various pH-responsive polymer systems have been studied, such as liposomes [21], micelles [22,23], nanoparticles [24], and nanogels [25,26,27]. The pH-responsive polymers can undergo phase transition at a specific pH values and direct spontaneous assemblies [28,29,30,31]. Polyelectrolytes, which contain weak acidic or basic groups, have been exploited as pH responsive polymers, inspiring advances in polymer research. Polybases, like poly(2-(diethylamino)ethyl methacrylate) (PDEAEMA), poly(4 or 2-vinylpyridine) (PVP), and poly(vinyl imidazole), were developed for biomedical applications in gene transfer, bio-imaging, and controlled drug release, due to their excellent physicochemical properties [32,33,34]. The amine group, pyridine, and imidazole group in polymer chains can accept protons under acidic condition and release them in basic environments to induce phase transition [35,36]. For instance, PDEAEMA dissolves in acidic aqueous solution due to the electrostatic repulsions between the protonated amine groups, but undergoes a precipitation above pH 7.5, caused by the hydrophobic effect of substituted ethyl group. However, a challenging issue faced by these widely used polybases is that their phase transition values from the solution state to the precipitation state are usually fixed. Therefore, they are less accurate when targeting cell organelles with different pH microenvironments. The broad pH transition range of these polybases also limits the increase in their responsive property. To develop responsive polymers with narrow transition pH ranges, Armes et al. [37] reported a novel polybase system, poly(2-(diisopropylamino)ethyl methacrylate) (PDPAEMA), with a phase transition at pH 6.3 and a sharp pH transition, providing a method by which to prepare ultra-sensitive pH-responsive polymers.

In this work, we developed a series of novel reversible pH-responsive (co)polymers with a fine-tunable pH transition value. These polymers were synthesized using a free radical polymerization method with tertiary amine-based monomers, 2-(dibutylamino)ethyl methacrylate and 2-(dimethylamino)ethyl methacrylate. At low pH, the (co)polymers were in the unimer state due to the electrostatic interaction and hydrophilicity of the protonated tertiary amine units. At high pH, the stronger hydrophobicity of the alkyl groups on the deprotonated amines led to (co)polymer aggregation. All the (co)polymers showed rapid phase separation at their p*K*_a_, and the pH transition range can be controlled within a very narrowly defined value.

## 2. Results

### 2.1. Synthesis of the (Co)polymers

**Figure 1:**
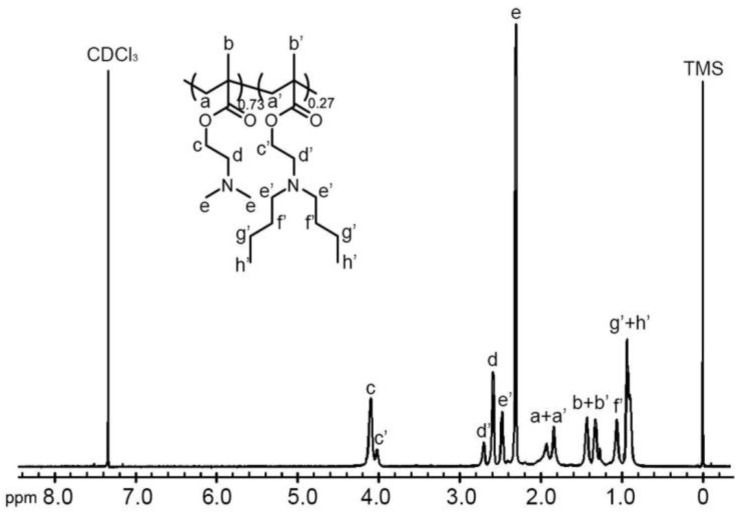
Proton nuclear magnetic resonance (^1^H-NMR) spectrum of poly(2-(dimethylamino)ethyl methacrylate)-*co*-2-(dibutylamino)ethyl methacrylate (P(DMAEMA_0.73_-*co*-DBAEMA_0.27_)) in CDCl_3_.

A series of random copolymers poly(2-(dimethylamino)ethyl methacrylate)-*co*-2-(dibutylamino)ethyl methacrylate (P(DMAEMA-*co*-DBAEMA)) were obtained by conventional free radical polymerization with varying feed ratios of the monomers containing different tertiary amine groups. Comparing the signal integrals of the proton peaks at 2.3 ppm and at 2.5 ppm (Figure 1, Figures S1–S4), the compositions of the copolymers could be determined, as summarized in Table 1. The molar ratios of the monomer units in the copolymers were relatively close to the feed ratios. Due to the methacrylic acid ester structure of the two monomers, random sequences of two monomer units were expected in the copolymer chains.

### 2.2. pH Titration of Different Copolymers and Measurement of pK_a_

**Figure 2:**
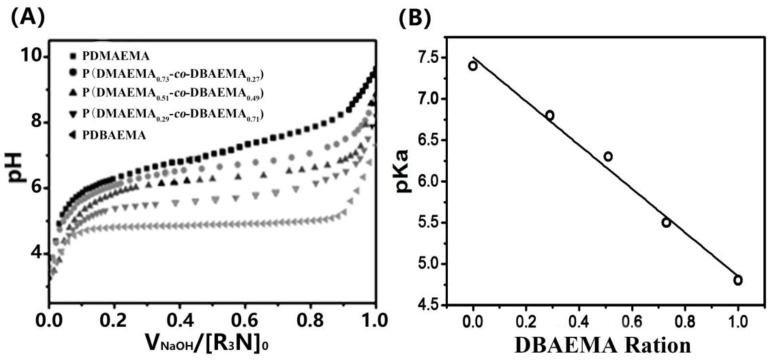
(**A**) pH titration curves of (co)polymers. The volumes of NaOH (V_NaOH_) were normalized to the initial amount of amine ([R_3_N]_0_ in mmol); (**B**) pKa values of the tertiary amine-based polymers as a function of DBAEMA ratio.

For pH-responsive polymers, the p*K*_a_ value is a critical parameter that can be evaluated through titration experiments. Figure 2A shows the potentiometric titration curves for 0.5 mg/mL (co)polymer solutions. NaOH solution was added to neutralize the protons. Potentiometric titration curves were produced for (co)polymers by plotting the solution pH against the volume of added NaOH. Starting from pH ~3, the addition of small aliquots of NaOH would neutralize the free protons and increase the solution pH. The pH value of PDMAEMA increased continuously with higher NaOH content, whereas for the other (co)polymers, especially PDBAEMA, titration curves were obtained with a plateau at the polymer buffering region. This indicates that the added NaOH was consumed during the deprotonation of the tertiary amine groups on the polymer pendent chain, which corresponds to the phase transition of the polymer. With the completion of the process, further addition of base increased the solution pH rapidly. For each sample, the p*K*_a_ values were calculated as the pH halfway between the two equivalence points in the titration curve. With increasing DBAEMA monomer amounts from 0% to 100%, as shown in Figure 2B, the average p*K*_a_ values of the polymers decreased from 7.4 to 4.8, which was attributed to the hydrophobic property of butyl on amine. An approximately linear relationship was observed between the p*K*_a_ values and DBAEMA monomer molar ratios of the (co)polymers, which indicates that the p*K*_a_ of the pH sensitive polycations can be controlled easily and precisely by adjusting the hydrophobic properties of the pendent group.

### 2.3. Phase Transition Behaviors of the Fine-Tuning pH Responsive Polymers

**Figure 3:**
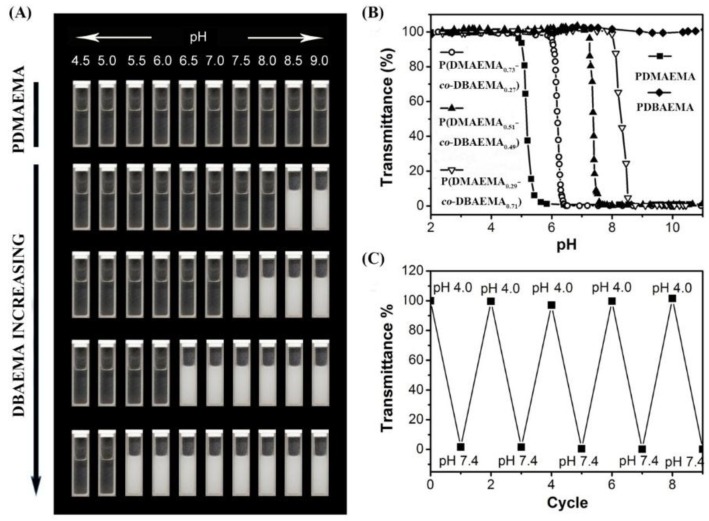
(**A**) Optical images of (co)polymer solutions at different pH. (**B**) The transmittance curves of (co)polymer solutions with increasing pH values. (**C**) The pH reversibility study of PDBAEMA solution with cycles between pH 4.0 and pH 7.4.

The tenability of the (co)polymers’ pH-responsive properties were evaluated by transmittance experiments. Figure 3A,B show the optical images and transmittance changes of the polymer solutions as NaOH was added from initial acid solutions, where all of the polymers were completely dissolved. Over the entire pH range in the measurements, the PDMAEMA solution remained clear with a transmittance around 100%, which was likely due to the hydrophilicity of the dimethyl amino groups, in good agreement with the titration experiment. However, with the incorporation of DBAEMA, the turbidity of the polymer solutions increased sharply at critical pH values due to the aggregation of the polymer chains. The phase transition pH range decreased with the increasing ratio of DBAEMA. Compared to conventional pH responsive materials, the DBAEMA-based polymers displayed a much sharper pH-dependent phase transition, where the transmittance from 100% to 0% was limited to 0.3 pH, indicating that only double the proton concentration could lead to complete turbidity of the copolymers in aqueous solutions. Furthermore, the reversibility of the pH-responsive tertiary amine-based polymers was confirmed by the PDBAEMA transmittance measurement. We also analyzed the polymer solution during cyclic tests between pH 4.0 and pH 7.4. As shown in Figure 3C, the dissolution and aggregation of the polymer solutions are fully reversible for at least five cycles, which indicates the novel pH sensitive materials can be used repeatedly.

**Figure 4:**
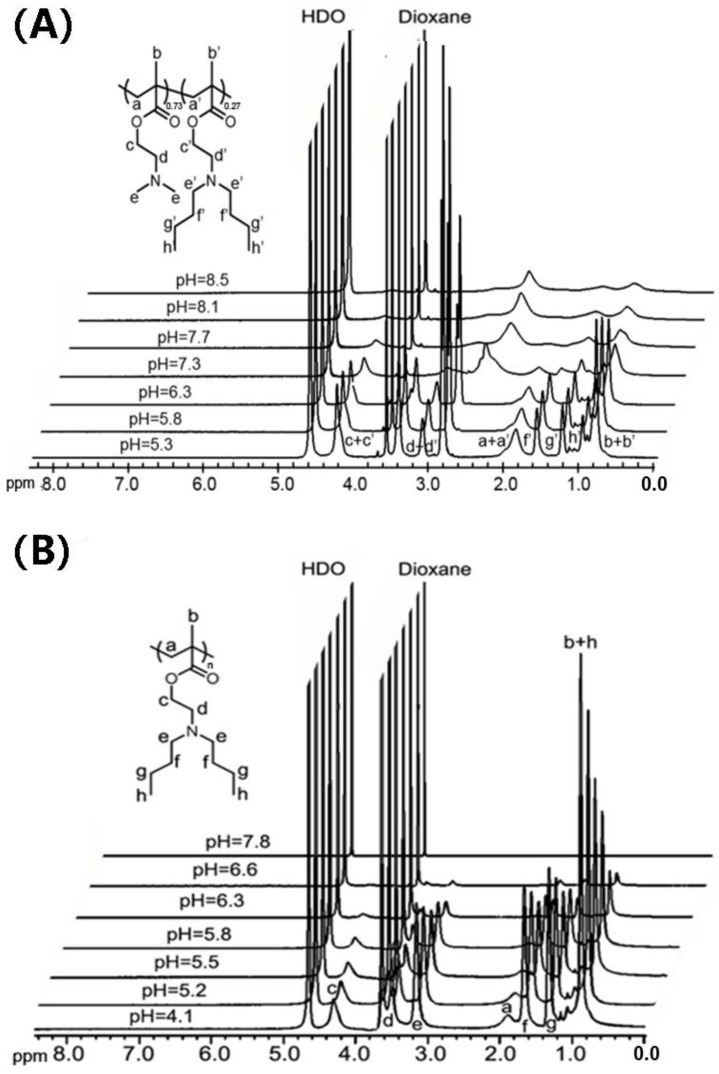
^1^H-NMR spectra of (**A**) P(DMAEMA_0.73_-*co*-DBAEMA_0.27_) and (**B**) PDBAEMA at different pH in D_2_O.

Proton nuclear magnetic resonance spectroscopy (^1^H-NMR) was also employed to investigate the effect of pH on the phase transition behaviors of the polymers in aqueous solution. PDBAEMA and P(DMAEMA_0.73_-*co*-DBAEMA_0.27_) were investigated as a function of pH in order to compare the aggregation behavior of the polymers with different tertiary amine groups. 1,4-Dioxane, with a single peak at 3.7 ppm, was used as the internal standard, as its intensity remained almost the same before and after the polymer underwent a phase transition. The spectra of the PDBAEMA and P(DMAEMA_0.73_-*co*-DBAEMA_0.27_) were recorded in the pH range of 4.1 to 8.5, and Figure 4 shows the ^1^H-NMR spectra of the polymers in D_2_O with the tertiary amines in different ionization states. At pH 4.1 and 5.3, the amine groups were protonated; both PDBAEMA and P(DMAEMA_0.73_-*co*-DBAEMA_0.27_) were dissolved in the deuterated solution and proton resonance peaks for the segments of each polymer were easily visualized. However, as pH increased from 5.5 to 6.6, the signal intensities of the PDBAEMA dramatically decreased and disappeared at pH 7.8, when PDBAEMA became completely deprotonated. The suppressed resonances of the polymer were due to the limitation of the motion of the polymer chain, which can be attributed to the phase transition behavior driven by the hydrophobic effect. For P(DMAEMA_0.73_-*co*-DBAEMA_0.27_), although the signal integrations of the polymer signals decreased gradually when the pH increased above 6.3, the peaks of DMAEMA segments were still observable even at pH 8.5. This drastic difference between PDBAEMA and P(DMAEMA_0.73_-*co*-DBAEMA_0.27_) spectra can be attributed to the dehydration behaviors of the polymers upon neutralization. The hydrophobic effect involving the butyl groups on the PDBAEMA pendent segments enabled the polymer chains to pack densely in aqueous solution, thus leading to more efficient dehydration and a sharper phase transition.

## 3. Materials and Methods

### 3.1. Materials

2-(dibutylamino) ethanol was purchased from Alfa Aesar Company (Haverhill, MA, USA) and 2-(dimethylamino)ethyl methacrylate (DMAEMA) was purchased from Sigma-Aldrich (St. Louis, MO, USA). CDCl_3_, D_2_O, and 1,4-Dioxane were purchased from Acros Co. (Beijing, China) and used as received. Tetrahydrofuran (THF) was freshly purified by distillation over sodium prior to use. 2,2-Azoisobutyronitrile (AIBN) was recrystallized three times from methanol. Other solvents and reagents were purchased from Beijing Chemical Reagent Co. (Beijing, China) and used as received.

### 3.2. Synthesis of the DBA Monomer

The tertiary amine-based methacrylate monomer 2-(Dibutylamino)ethyl methacrylate (DBAEMA) was synthesized by following a previously reported method [38]. 2-(dibutylamino)ethyl ethanol (17.3 g, 0.1 mol), triethylamine (10.1 g, 0.1 mol), and inhibitor hydroquinone (0.11 g, 0.001 mol) were dissolved in 100 mL THF and placed in a three-neck flask. To this solution, methacryloyl chloride (10.4 g, 0.1 mol) was added dropwise with constant stirring. The resulting solution was refluxed in THF for 2 h and then filtered to remove the precipitated white triethylamine-HCl salts with completion of the reaction. After removing THF solvent by rotary evaporator, the resulting residue was distilled in vacuo (83–87 °C at 0.05 mm Hg) as a colorless liquid. The obtained DBAEMA monomer was characterized by ^1^H-NMR. ^1^H-NMR (TMS, CDCl_3_, ppm): 6.09 (br, 1H, C*H*H=C(CH_3_)-), 5.55 (br, 1H, CH*H*=C(CH_3_)-), 4.19 (t, *J* = 6.3 Hz, 2H, -OC*H*_2_CH_2_N-), 2.73 (t, *J* = 6.3 Hz, 2H, -OCH_2_C*H*_2_N-), 2.46 (t, *J* = 7.6 Hz, 2H, -N(C*H*_2_CH_2_CH_2_CH_3_)_2_), 1.93 (s, 3H, CH_2_=C(C*H*_3_)-), 1.41 (m, 4H, -N(CH_2_C*H*_2_CH_2_CH_3_)_2_), 1.29 (m, 4H, -N(CH_2_CH_2_C*H*_2_CH_3_)_2_), 0.89 (t, *J* = 7.3 Hz, 6H, -N(CH_2_CH_2_CH_2_C*H*_3_)_2_), Yield: 56%.

### 3.3. Synthesis of the (Co)polymers

The P(DMAEMA-*co*-DBAEMA) copolymers, PDMAEMA, and PDBAEMA were prepared by free radical polymerization in THF with varying DBAEMA to DMAEMA molar feed ratios of 0:4, 1:3, 2:2, 3:1, and 4:0, respectively. Typical procedures employed for the polymerization were as follows. Monomers were dissolved in THF at a total concentration of 0.10 g/mL, to which azodiisobutyronitrile (AIBN) (1.0 mol% relative to monomers) was added as a free radical initiator. After three cycles of freeze-thaw to thoroughly remove oxygen, the tube was sealed under reduced pressure and the polymerization was performed at 60 °C for 24 h. The polymers were purified by precipitation from diethyl ether twice, collected by filtration, and dried under vacuum to obtain white powders. The molecular weights and the composition of the (co)polymers were determined by gel permeation chromatography (GPC) and ^1^H-NMR, respectively.

### 3.4. Characterization of the (Co)polymers

^1^H-NMR spectra of the monomers and (co)polymers were recorded in CDCl_3_ on a Varian-600 MHz spectrometer with tetramethylsilane (TMS) as the internal reference. The molecular weight and molecular weight distributions of the (co)polymers were measured with gel permeation chromatography (GPC) equipment consisting of WGE3010 pump, 3010 refractive index detector, and WGE Styra gel columns. The temperature of the columns was 35 °C and THF was used as an eluent at a flow rate of 1 mL/min. A series of narrowly dispersed polystyrene samples were used as standards and Millennium 32 software was used to calculate the molecular weight and polydispersity.

### 3.5. pH Titration

The p*K*_a_ values of different (co)polymers were detected by pH titration. When the prepared polymer was dissolved in 0.1 N HCl to reach a final concentration of 5–10 mg/mL, the pH titration experiment was performed by adding small volumes (50–100 μL increments) of 0.1 N NaOH solution under stirring. The pH values of the solution were measured continuously using a Sartorius (Germany) pH meter with a microelectrode.

### 3.6. Turbidity Measurements by UV/Vis Spectroscopy

The pH-dependent phase transition of the polymers was determined by the turbidity of the polymer solutions as a function of pH. The transmittance of the 1 mg/mL (co)polymer solutions in 0.1 N HCl were measured at 500 nm through a 1 cm quartz cell on a Shimadzu 2550 ultraviolet (UV)-vis spectrometer. To adjust the pH, 0.2 N NaOH was added, and the final volumes of the polymer solutions increased about 10% compared with initial volumes. The pH values were monitored with a digital internal pH meter. Polymer-free deionized (DI) water was used as a reference.

### 3.7. ^1^H-NMR Measurements of pH-Dependent Phase Transition

P(DMAEMA_0.73_-*co*-DBAEMA_0.27_) and PDBA were first dissolved in the deuterated buffers at a concentration of 10 mg/mL, and 1,4-dioxane was used as an internal standard. To adjust the pH value, 0.1 N NaOD deuterated solution was added and the Varian Mercury Plu600 MHz NMR spectrometer was used to record the ^1^H-NMR spectra at different pH.

## 4. Conclusions

A series of novel pH-responsive polymers based on tertiary amine functional groups were developed by easy free radical copolymerization. The p*K*_a_ values of the polymers were precisely tuned by adjusting the feed ratio between the two monomers, DMAEMA and DBAEMA. The phase transition of polymers occurred in narrow pH ranges, which was demonstrated by transmittance and ^1^H-NMR detection. Below p*K*_a_, the positive charges of the protonated amine groups maintained the solubility of the polymers in aqueous solution, whereas when pH was greater than p*K*_a_, the hydrophobic butyl groups on neutralized PDBAEMA segments rapidly induced the aggregation and precipitation of the polymers. Compared to widely used PDMAEMA and PDEAEMA, the developed polymers with PDBAEMA segments displayed much sharper pH transition ranges. The finely tunable transition pH values mean the polymers are a promising platform for drug delivery and biomedicine applications, where the encapsulated drugs at physiological pH would be triggered to release in acidic microenvironments.

## Figures and Tables

**Table 1:** Characterization of (co)polymers.

Copolymer	DMA Content		Yield (%)	*M_n_*^b^ (×10^4^)	*M_w_*^b^ (×10^4^)	PDI	p*K*_a_^c^
	In Feed	In Polymer ^a^					
PDMAEMA	100	100	73	1.21	1.85	1.53	7.4
P(DMAEMA_0.73_-*co*-DBAEMA_0.27_)	75	73	67	1.06	1.47	1.39	6.8
P(DMAEMA_0.51_-*co*-DBAEMA_0.49_)	50	51	62	1.01	1.84	1.82	6.3
P(DMAEMA_0.29_-*co*-DBAEMA_0.71_)	25	29	65	1.13	2.03	1.80	5.5
PDBAEMA	0	0	56	1.40	2.59	1.85	4.8

^a^ Percent molar ratio determined by hydrogen nuclear magnetic resonance (^1^H-NMR); ^b^ Determined by gel permeation chromatography (GPC) in tetrahydrofuran (THF) with polystyrene standards; ^c^ Determined by titration during the heating process.

## Supplementary Materials

The following are available online.
