# Supplementary material for: Ultrasensitive (Co)polymers Based on Poly(methacrylamide) Structure with Fining-Tunable pH Responsive Value
Source: Molecules. 2018 Jul 27;23(8):1870. doi: 10.3390/molecules23081870 (PMC6222650; doi:10.3390/molecules23081870)
Supplement: Supplementary file 1 [file molecules-23-01870-s001.pdf]

Electronic Supplementary Material (ESI) for Molecules.

SUPPORTING INFORMATION

Ultrasensitive (co)polymers based on Poly(methacrylamide) structure with fining-tunable pH responsive value

Haiming Fan [a] , Po Li [b] , Wei Li [b], Hui Li [a], Xiaonan Huang [* b]

a. School of Petroleum Engineering, China University of Petroleum (East China), Qingdao 266580, Shandong Province, PR China.

b. Department of Chemistry, Capital Normal University, 105 West 3rd Ring North Rd, Beijing 100048, PR China.

*** To whom correspondence should be addressed:**

Dr. Xiaonan Huang

E-mail: huangxn@cnu.edu.cn (X.N. Huang)

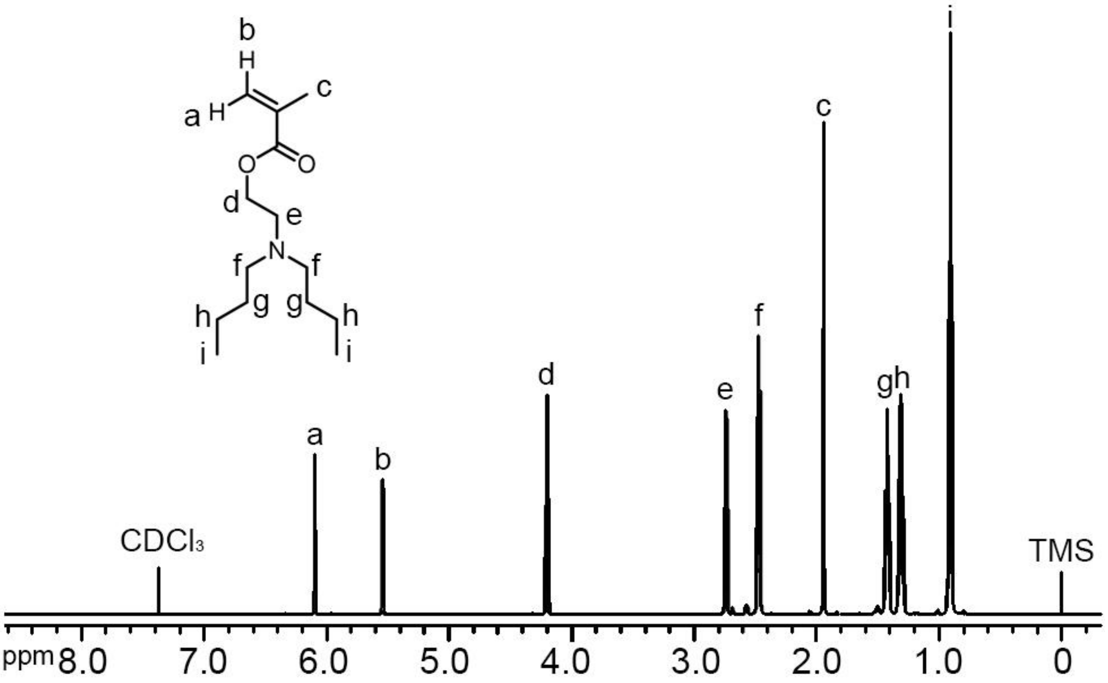

Fig. S1. ^{1}H NMR spectrum of DBAEMA in $CDCl_3$.

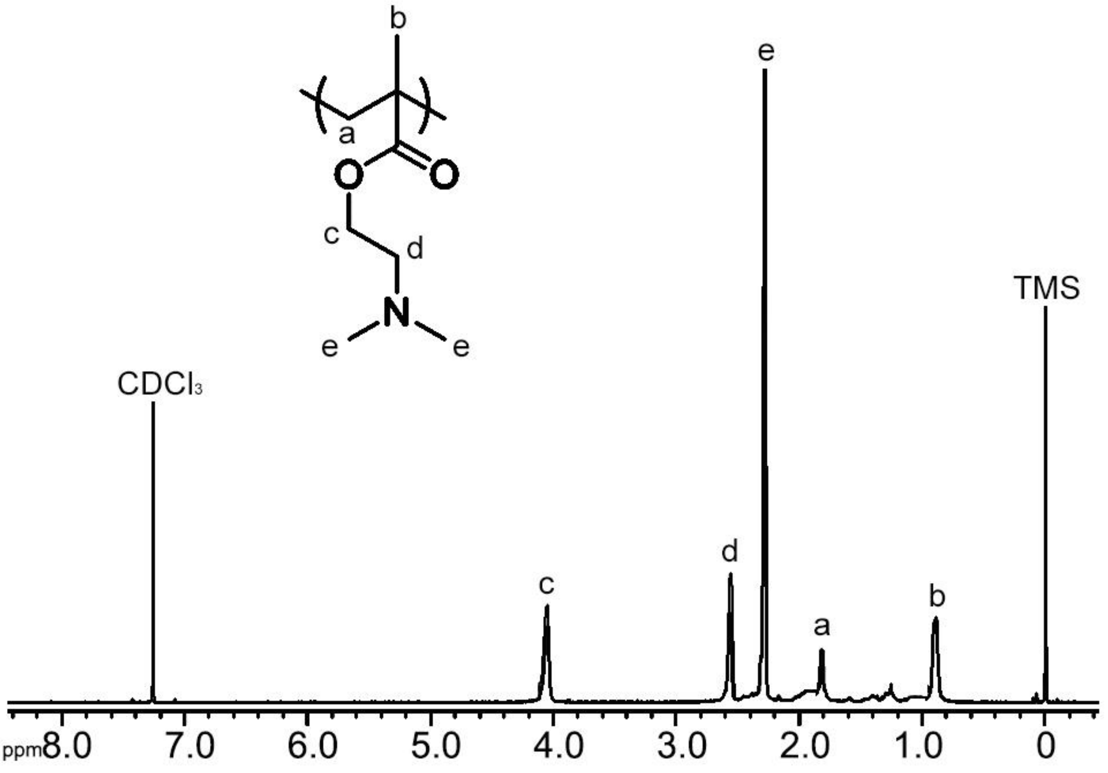

Fig. S2. ^{1}H-NMR spectrum of PDMAEMA in $CDCl_3$.

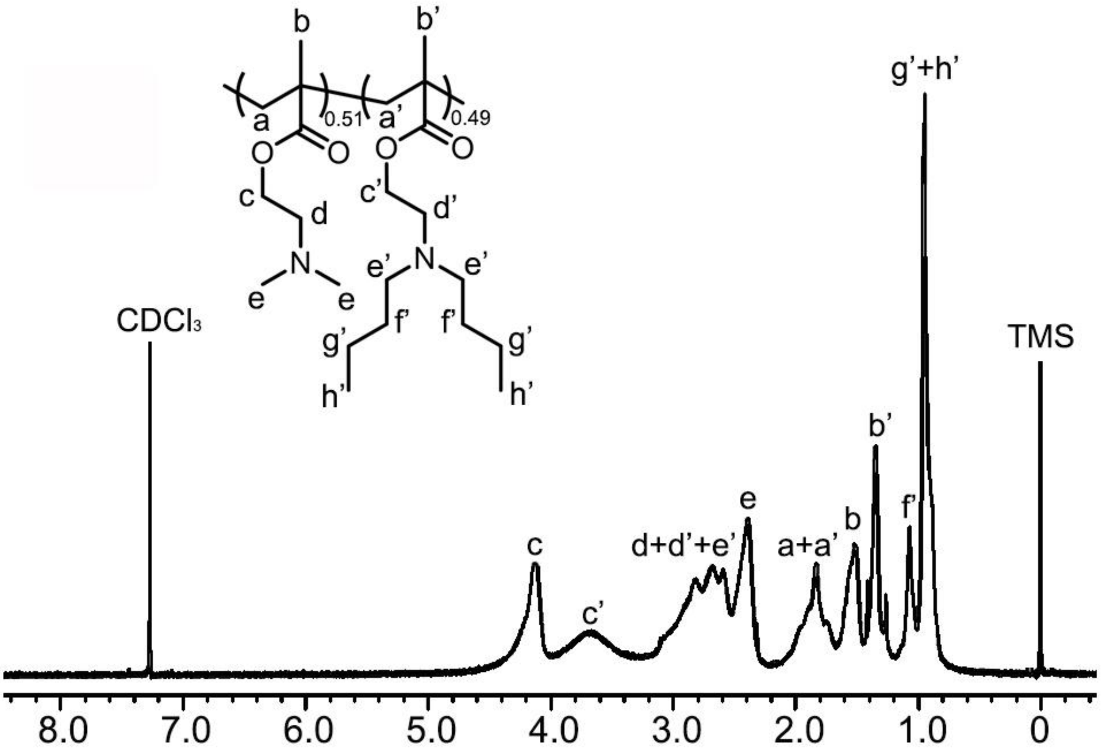

Fig. S3. ^{1}H-NMR spectrum of P(DMAEMA$_{0.51}$-*co*-DBAEMA$_{0.49}$) in $CDCl_3$.

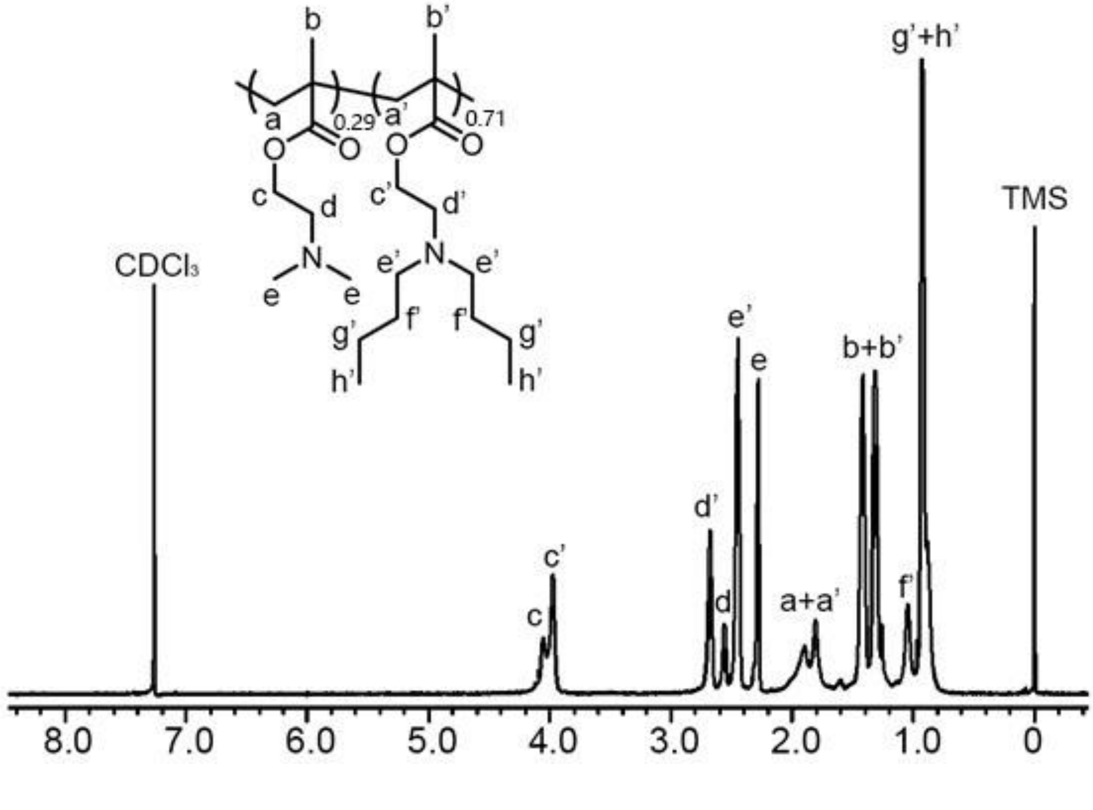

Fig. S4. ^{1}H-NMR spectrum of P(DMAEMA$_{0.29}$-*co*-DBAEMA$_{0.71}$) in $CDCl_3$.

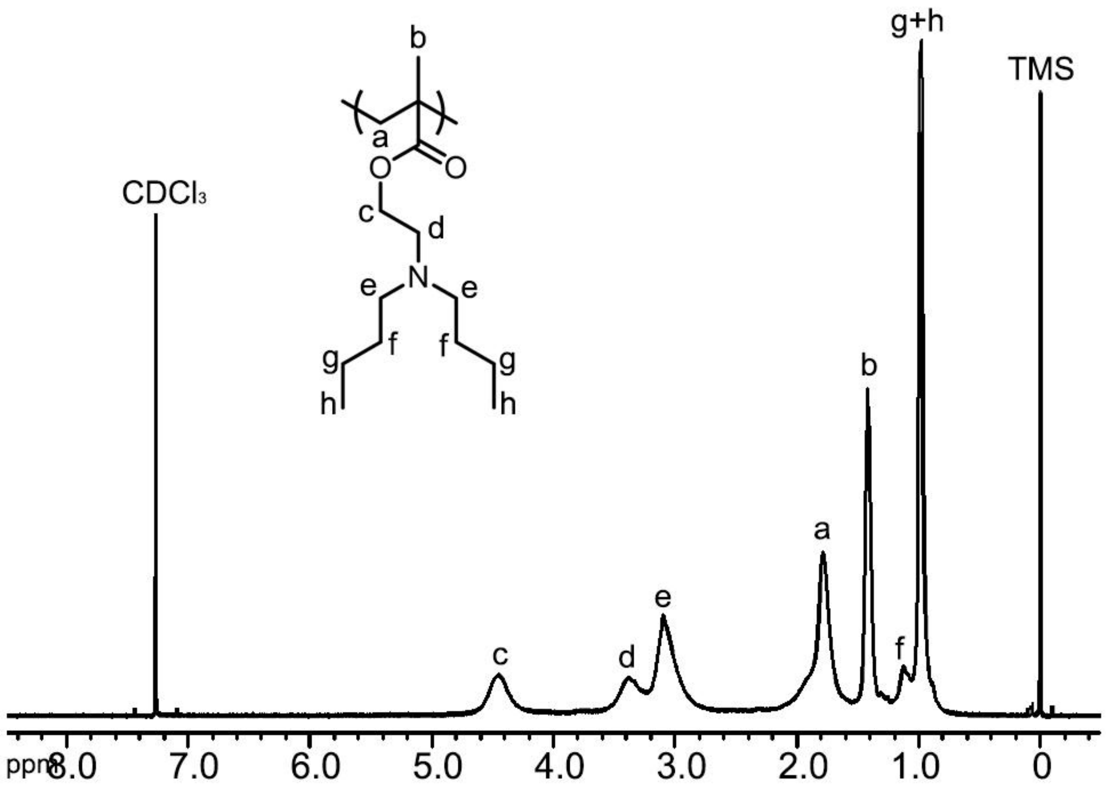

Fig. S5. ^{1}H-NMR spectrum of PDBAEMA in $CDCl_3$.

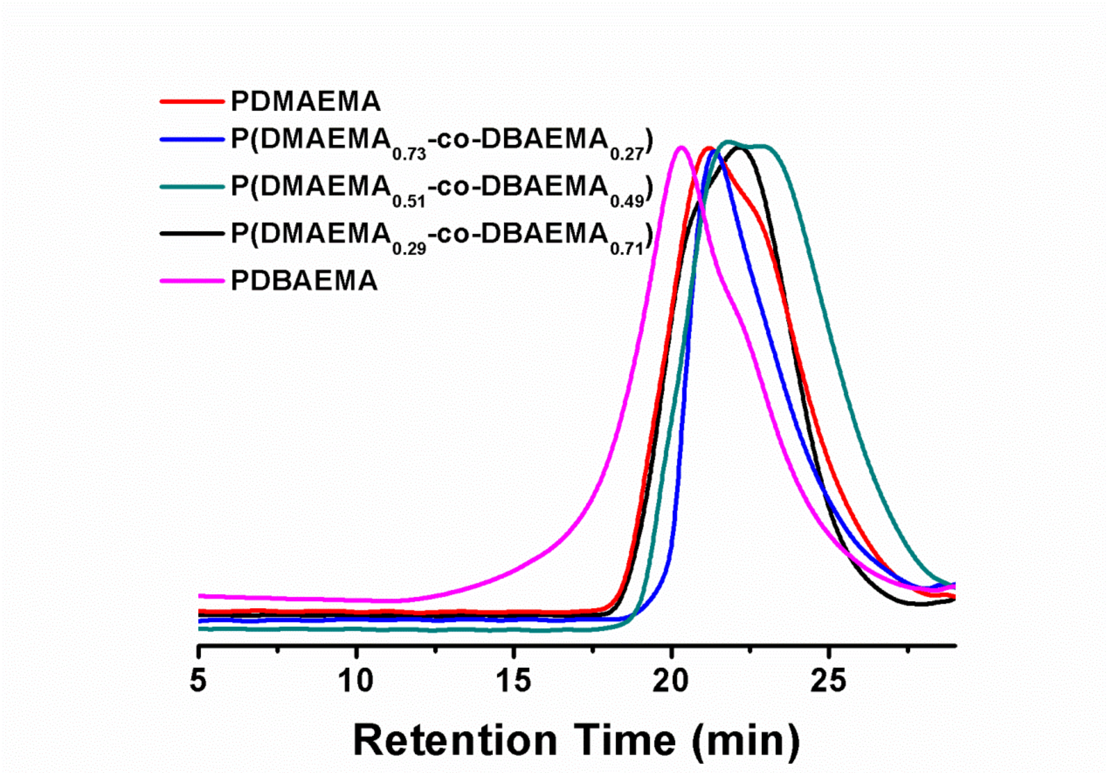

Fig. S6. GPC traces of (co)polymers.

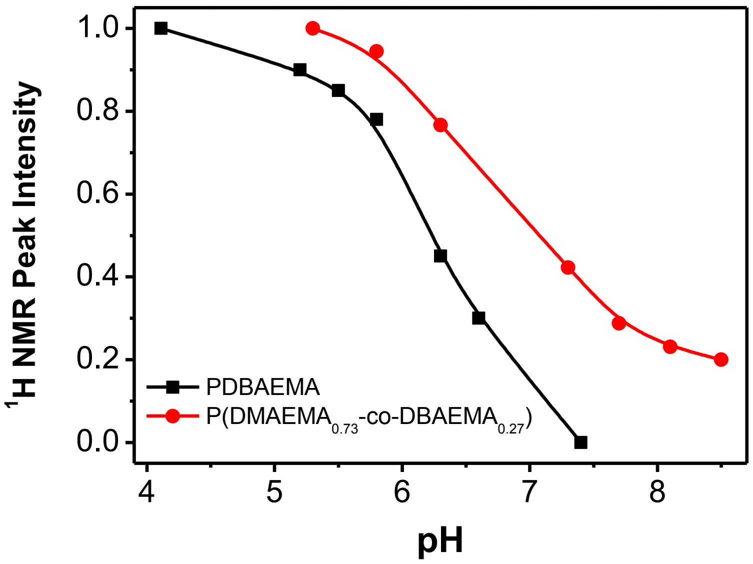

Fig. S7. ^{1}H NMR peak intensity of peak e vs pH for PDBAEMA and P(DBAEMA$_{0.73}$–*co*-DBAEMA$_{0.27}$)

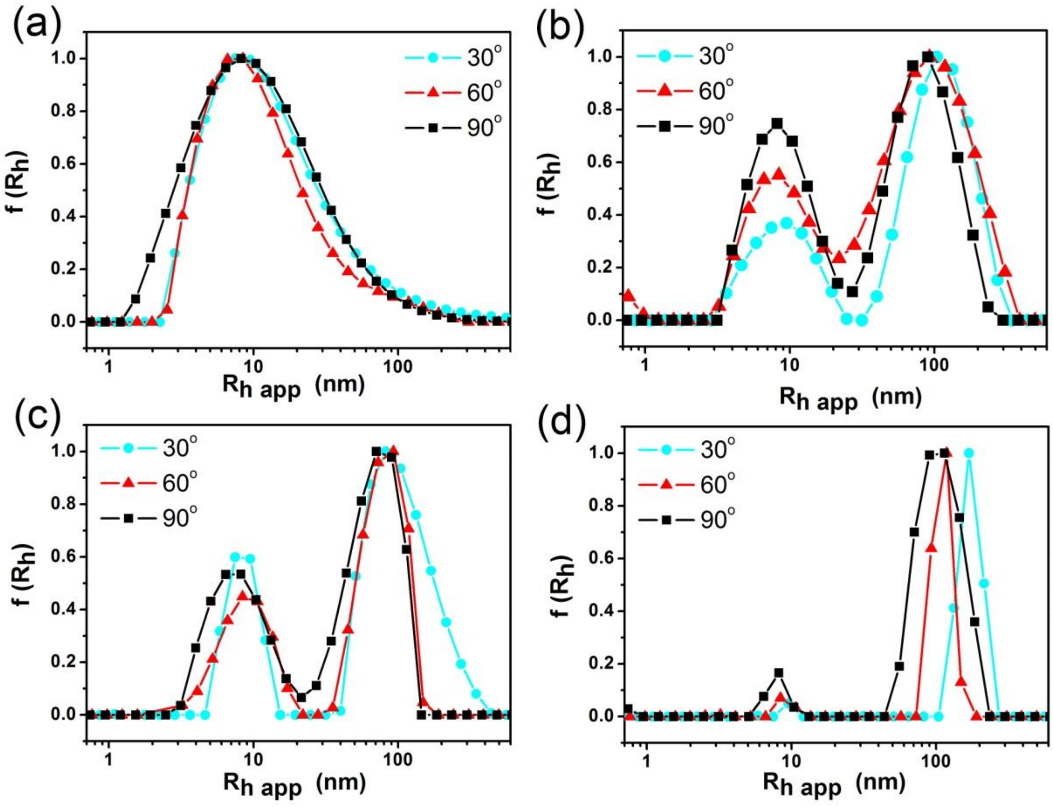

Fig. S8. Contin analysis of PDBAEMA at different pH (a)pH=2.09；(b)pH=3.20；(c)pH=3.39；(d)pH=4.55

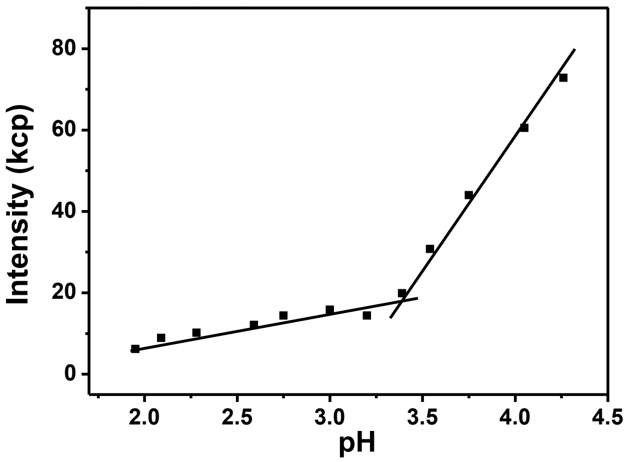

Fig. S9. The dependence of Light scattering intensity with pH(Detection Angle 30°).
